# Streamlining Venepuncture: Reducing Time Wasted and Improving Patient Care on a Specialist Eating Disorder Unit (SEDU)

**DOI:** 10.1192/bjo.2025.10378

**Published:** 2025-06-20

**Authors:** Leah Jacob, John Adam, Robert Hyder-Wilson, Ewa Zadeh

**Affiliations:** Springfield University Hospital, London, United Kingdom

## Abstract

**Aims:** Specialist eating disorder units (SEDUs) are unique among psychiatric units, in that there is a high incidence of electrolyte derangements due to the pathophysiology of refeeding. This requires careful monitoring of blood parameters with frequent venepuncture which often can be difficult due to strict mealtimes and post meal supervision as well as required group attendance in the SEDU.

Audit data demonstrated that the medical team spent about 3 hours every day and therefore 15 hours per week attempting to obtain blood samples from patients due to inefficient processes. Patients were often unavailable due to other commitments and so a maximum of 4 blood tests were obtained each day.

The primary aim of the project was to reduce the amount of time spent obtaining blood samples on the SEDU. Our secondary aims were to reduce patient uncertainty around venepuncture and to improve patient satisfaction.

**Methods:** A ‘phlebotomy clinic’ was implemented twice a week to replace daily venepuncture. The clinic was made up of 5-minute appointments and scheduled based on the published weekly ward schedule to avoid any protected mealtimes and group activities. The clinics took place at the start of the week to allow more time for results to be analysed and actioned.

Universal consent was gained from our patient group by discussion at the community meeting. It was agreed that a list of appointments would be published on the notice board and patients would be reminded about the clinic at morning check in.

The amount of time spent obtaining blood samples was self-reported by doctors at the end of the week and patient satisfaction was graded using a qualitative questionnaire.

**Results:** Implementation of the phlebotomy clinic saved 13 hours of time per week. Over three separate phlebotomy clinics, the average time spent obtaining blood samples was 35 minutes with 25 minutes of admin time and an average of 6 blood samples were taken at each clinic. Patient adherence to appointment times varied between clinics with a range of 66–100% adherence and this impacted the efficiency of the clinic. Patient questionnaires demonstrated that 100% of patients preferred the new format.

**Conclusion:** We concluded that the implementation of a formal ‘phlebotomy clinic’ significantly improved efficiency of venepuncture on the SEDU allowing more time to be spent engaging in other aspects of patient care. In addition, patient satisfaction improved and we believe that this in turn can greatly benefit the therapeutic relationship.

